# Analysis of the global trends and causes of self-harm due to high temperature: a global level ecological study

**DOI:** 10.1265/ehpm.25-00057

**Published:** 2025-07-03

**Authors:** Jingjie Ma, Xingchao Zhang, Sanqian Chen, Siyu Zhou, Jing Ding, Yuting Deng, Jiakang Hu, Fang Wang, Yuanan Lu, Songbo Hu

**Affiliations:** 1 School of Public Health, Jiangxi Medical College, Nanchang University, Nanchang, Jiangxi, China; 2 Jiangxi Provincial Key Laboratory of Disease Prevention and Public Health, Nanchang University, Nanchang, Jiangxi, China; 3 Science Department of Jiujiang University, Jiujiang, Jiangxi, China; 4 Office of Public Health Study, University of Hawaii at Manoa, Honolulu, Hawaii 96822, USA

**Keywords:** Climate change, Suicide, Self-harm, High temperature, GBD

## Abstract

**Background:**

High temperatures are known to be associated with an increased risk of self-harm, but the influence of demographic changes and country-level indicators on the burden of heat-related self-harm remains unclear. This study examined the key factors driving changes in self-harm mortality linked to high temperatures and explored their impact at the country level.

**Methods:**

This is an ecological study that analyzes data from the 2021 Global Burden of Disease (GBD) study, the World Bank, and the Climate Research Unit (CRU) were analyzed. Decomposition analyses were used to identify key factors driving changes in high temperature-related self-harm mortality between 1990 and 2021. A panel data model assessed the impact of national indicators on heat-related self-harm mortality.

**Results:**

In 2021, 14,885 deaths globally were attributed to heat-related self-harm, a 41.94% increase from 1990, with low-middle SDI regions accounting for 47.84% of these deaths. While the global death rate from heat-related self-harm declined slightly over this period, South Asia and low-middle SDI regions contributed most to the decline. However, population aging exacerbated mortality rates. Demographic and meteorological factors were also linked to heat-related self-harm.

**Conclusion:**

The global decline in heat-related self-harm mortality is largely driven by reductions in females, low-middle SDI regions, and South Asia. However, population aging and growth in these regions have added to the mortality burden, slowing the overall decline. Factors such as population density are also associated with heat-related self-harm. Targeted measures are needed to mitigate heat-induced self-harm more effectively in future.

## 1. Introduction

Self-harm refers to the deliberate infliction of physical harm on oneself, resulting in injury or death [1]. It is a significant predictor of suicide [2]. In the *Comprehensive Mental Health Action Plan 2013–2030* [3], member states of the World Health Organization (WHO) committed to reducing national suicide rates by one-third by 2030. Despite progress through national interventions, substantial efforts are still required to achieve this goal.

Self-harm arises from a complex interplay of factors, including individual elements like genetics, physical and psychological conditions, and lifestyle, as well as broader environmental influences, urbanization patterns, and national socio-demographic characteristics [4]. Among these, the link between high environmental temperatures and self-harm is well established, with climate warming increasingly becoming a global crisis [5]. In 2021, nearly 2% of global self-harm deaths (1.99%) were attributed to high temperatures. Extreme heat exacerbates social and environmental risks, negatively impacting mental and physical health, leading to mental illness, or worsening pre-existing conditions, which may culminate in self-harming behaviors [6].

The relationship between air pollution and self-harm remains a subject of debate. Some studies have identified associations between air pollutants such as O_3_, PM_2.5_, and SO_2_ and self-harm, but findings across studies are inconsistent [7–11]. Similarly, urbanization’s impact on self-harm rates is contested. While some research suggests higher self-harm rates in urban areas [12], the correlation between urbanization levels and suicide rates has yielded mixed results [13, 14]. For example, an Italian study found a negative association between population density and self-harm in men, with no observed effect on women [15]. Conversely, a Northern Ireland cohort study found no link between population density and self-harm risk [16]. Given that countries and regions differ in climatic, economic, demographic, and cultural contexts, the factors influencing self-harm can vary significantly. Therefore, a comprehensive examination of self-harm in the context of high-temperature exposure, considering national-level factors, is crucial.

Previous studies on factors influencing self-harm have primarily focused on individual countries or regions, often leading to heterogeneous findings. While some research has utilized historical data to assess global trends in heat-related self-harm [17], these studies have not thoroughly examined the factors driving the burden of self-harm or analyzed the impact of demographic changes on its variation.

To address these gaps, we aimed to leverage the most recent data to explore two key research questions: How have changes in various dimensions of population structure influenced self-harm mortality due to high temperatures between 1990 and 2021? And what is the impact of meteorological factors, air pollutants, demographic characteristics, and socio-economic indicators on heat-related self-harm mortality at the national level, and how are these relationships shaped?

## 2. Methods

### 2.1 Data sources

This study is an ecological study, and our research data for this study were obtained from three publicly available databases. Specifically, information on the number of self-harm deaths and mortality rates linked to high-temperature risk in 204 countries from 1990 to 2021 was retrieved from the Global Health Data Exchange website (http://ghdx.healthdata.org/gbd-results-tool). This data is part of the Global Burden of Disease (GBD) study, a comprehensive and systematic investigation. Self-harm was defined according to the International Classification of Diseases, Tenth Revision (ICD-10), using the codes X60–X64.9, X66–X84.9, and Y87.0 [18]. Additionally, high temperature is a level 3 risk factor in the GBD 2021, with high temperature defined as a daily mean temperature warmer than the theoretical minimum-risk exposure level (TMREL), the temperature with the minimum mortality for all included causes [19]. Methods of estimating self-harm attributable to high temperatures are described in detail elsewhere [20].

The GBD 2021 study employed a systematic methodology to quantify the impact of risk factor exposure on health outcomes [21]. Detailed explanations of the methods used by GBD to calculate the burden of high-temperature-induced diseases was described in a previous publication [22]. The Socio-Demographic Index (SDI) is a composite measure that reflects a nation’s average income level, average years of schooling, and fertility rate among women under 25 [23]. GBD 2021 classifies countries and regions into five levels based on the SDI (0–1): High (>0.81), high-middle (0.71–0.81), middle (0.62–0.71), low-middle (0.47–0.62), low (<0.47) [24]. Additionally, all the countries were grouped into 21 geographical regions based on socio-economic and geographical similarities.

Indicators of impact factors were obtained from two sources: a) the Climatic Research Unit’s Year-by-year Variation of Selected Climate Variables by Country (CRU CY) version 4.07 dataset (Climatic Research Unit - Groups and Centres (uea.ac.uk)) and b) the World Bank database (World Development Indicators | Data Catalog (worldbank.org)). The following four indicators were included in this study: a) Meteorological indicators - diurnal temperature range (°C), vapor pressure (hPa) and rain days (days); b) Air pollutant indicator - PM2.5 (µg/m^3^); c) Demographic indicators - population density (persons/km^2^), population growth (annual %), urban population growth (annual %) and urban population (% of the total population); d) Socioeconomic indicators - life expectancy at birth (years), GDP per capita growth (annual %), GDP per capita (constant 2015 USD), and number of physicians (per 1000 people). The meteorological indicators are derived from CRU dataset, and the other indicators were from the World Bank database. Due to insufficient data in some countries, 174 countries and regions from 1990 to 2021 were selected for this study to analyze the relationship between high-temperature-induced self-harm and these indicators.

### 2.2 Statistical analysis

Decomposition analyses were conducted to quantify the contribution of demographic changes and other factors to variations in heat-induced self-harm mortality over the past 30 years. Decomposition analysis involved converting a comparison of mortality between two populations into a analysis of mortality differences across two points in time. It provides insights into how changes in population structure and specific factors influence mortality trends over a given period. A negative contribution of a factor indicates that it reduces mortality, while a positive contribution suggests that it increases mortality. A negative or positive contribution of a factor simply indicates that the factor reduces or increases the change in mortality.

The decomposition method is derived as follows: let the crude mortality rate for self-harm attributable to high temperature in 1990 be CMR^1^, the crude mortality rate for self-harm attributable to high temperature in 2021 be CMR^2^, Δ be the difference between the above, C_k_ be the population composition of the five SDI regions, and M_k_ be the mortality rate for self-harm attributable to high temperature for the corresponding SDI region. Then:

$$\begin{array}{rl} \Delta & ={\mathrm{CMR}}^{2}-{\mathrm{CMR}}^{1}=\sum C_{k}^{2}M_{k}^{2}-\sum C_{k}^{1}M_{k}^{1}\\ & =\sum C_{k}^{2}\frac{\left(M_{k}^{2}+M_{k}^{1}\right)}{2}-\sum C_{k}^{1}\frac{\left(M_{k}^{2}+M_{k}^{1}\right)}{2}\\ & \;+\sum M_{k}^{2}\frac{\left(C_{k}^{2}+C_{k}^{1}\right)}{2}-\sum M_{k}^{1}\frac{\left(C_{k}^{2}+C_{k}^{1}\right)}{2}\\ & =\sum (C_{k}^{2}-C_{k}^{1})\frac{\left(M_{k}^{2}+M_{k}^{1}\right)}{2}+\sum (M_{k}^{2}-M_{k}^{1})\frac{\left(C_{k}^{2}+C_{k}^{1}\right)}{2} \end{array}$$

Demographic contributed value = $\sum (C_{k}^{2}-C_{k}^{1})\frac{\left(M_{k}^{2}+M_{k}^{1}\right)}{2}$ ,
Demographic contributed rate = Demographic contributed value/Δ × 100%;
SDI regional contributed value = $\sum (M_{k}^{2}-M_{k}^{1})\frac{\left(C_{k}^{2}+C_{k}^{1}\right)}{2}$ ,
SDI regional contributed rate = SDI regional contributed value/Δ × 100%.

A panel data model [25] was used to analyze factors associated with high-temperature-induced self-harm from 1990 to 2021. Variance inflation factors (VIF) were calculated to assess multicollinearity among the model’s independent variables. A VIF value greater than 10 indicates a high degree of multicollinearity.

Panel models are categorized as fixed effects models, random effects models and mixed effects models. If there are no significant differences between individuals and over time, a mixed-effects model can be used; otherwise, a fixed-effects model or a random-effects model is selected by the Hausmann test. A fixed effects model was used in this study in the form of a model:

$$y_{it}=\alpha +\alpha_{i}+\sum_{k=1}^{K}x_{itk}\beta_{k}+\mu_{it}$$

In this study, *y* denotes the number of deaths from heat-related self-harm, *i* (1, 2, …, 174) represents the number of countries and regions included in the analysis, *t* denotes the year, specifically 1990, 1991, …, 2021, *x* denotes the indicator of each impact factor, *K* denotes the number of explanatory variables, *β* is the regression coefficient, and *μ_it_* is the random error. When the p-value is less than 0.05, β > 0 indicates a positive effect and β < 0 indicates a negative effect.

The analysis was conducted in two steps: a) the raw indicators were analyzed using a panel data model to assess their individual impact on heat-related self-harm mortality, and b) the indicators were standardized [26] to eliminate the effects of scale, size, and directionality.

The method of standardization used as mentioned above is normalization, specifically scaling the data to a specific range, usually the interval [0, 1]. It can be done by scaling the data to a fixed interval through a linear transformation so that data of different magnitudes have similar scales. The specific formula is as follows:

$$x^{\prime }=\frac{x-\min(X)}{\max(X)-\min(X)}$$

Where *x*′ is the raw value of the influencing factor; min(*X*) is the minimum value of a single indicator in, max(*X*) is the maximum value of a single indicator; and *x*′ is the normalized indicator.

R software version 4.2.2 was employed for all data descriptions and analyses. The ‘plm’ package was used to analyze the panel data model. Outcomes were defined as statistically significant at P < 0.05.

## 3. Results

### 3.1 Description of death

**Fig. 1:**
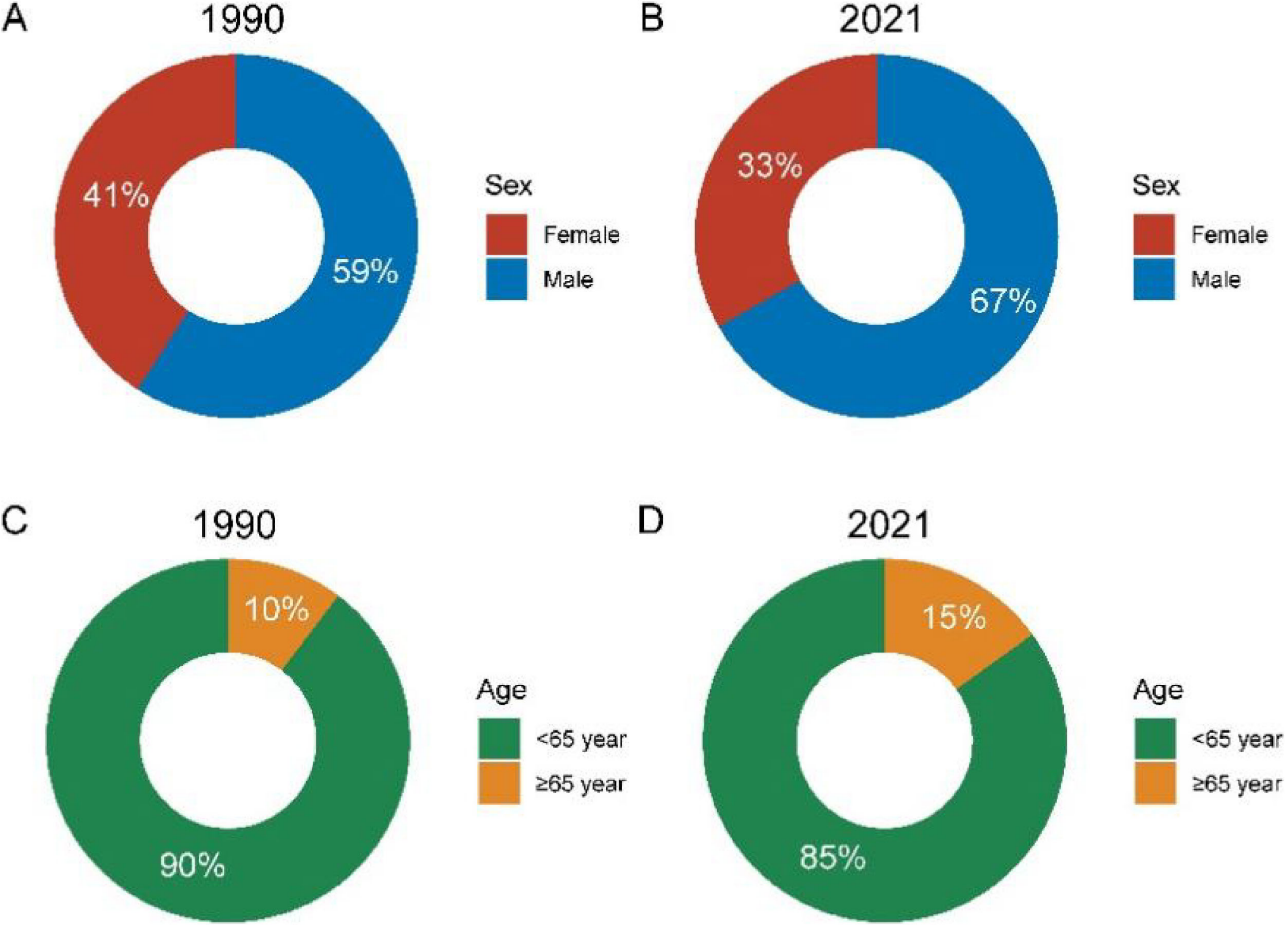
Distribution of heat-related self-harm deaths by sex and age, 1990 and 2021

**Fig. 2:**
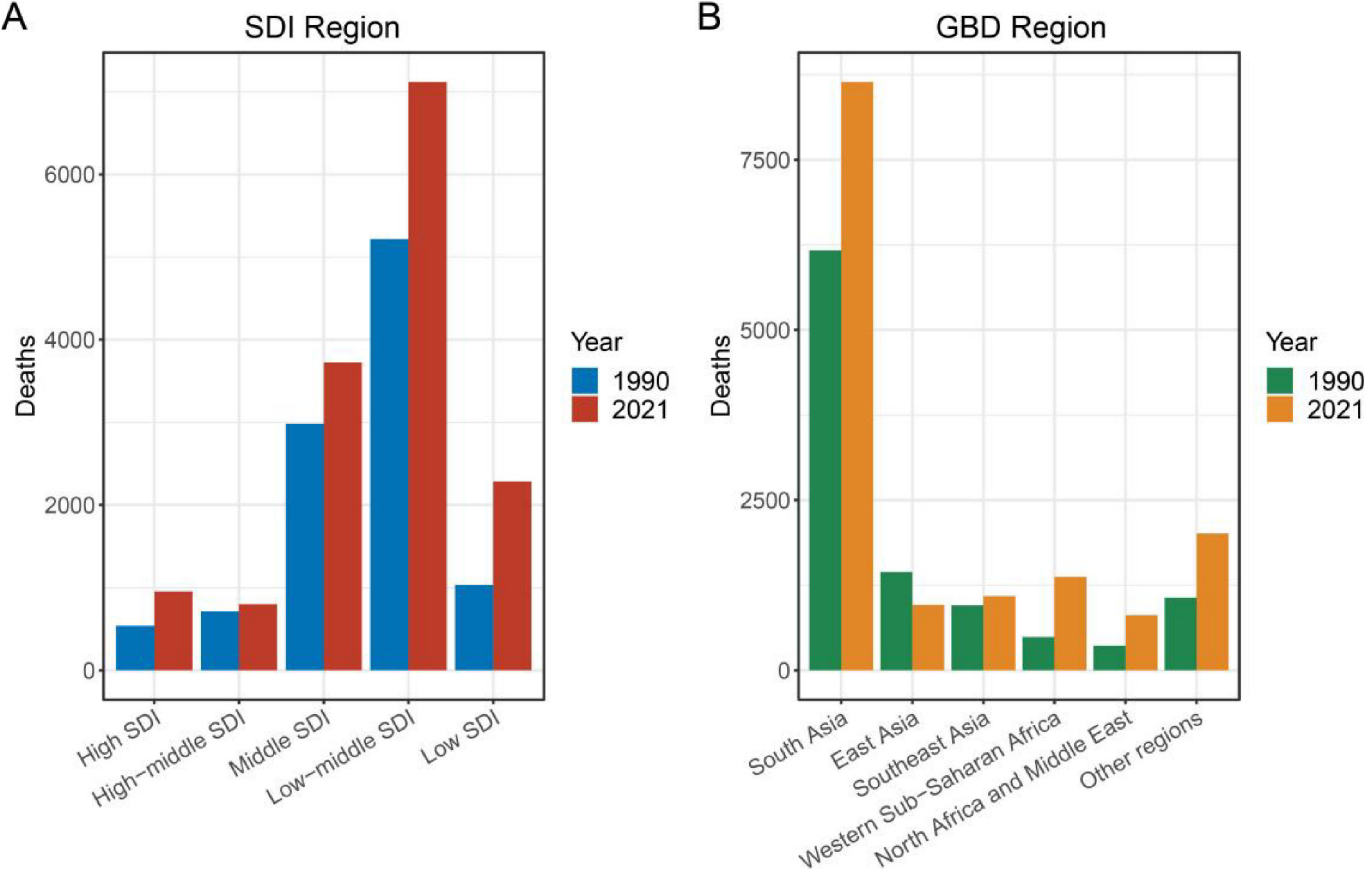
Distribution of heat-related self-harm deaths in SDI and GBD regions, 1990 and 2021

From 1990 to 2021, the number of global self-harm deaths related to high temperatures increased from 10,487 to 14,885. In terms of gender, the proportion of heat-induced self-harm deaths that were male increased from 59% in 1990 to 67% in 2021, and in terms of age, the proportion of heat-induced self-harm deaths that were in people aged 65 years and older increased from 10% in 1990 to 15% in 2021 (Fig. 1). Among the five SDI regions, the low-middle SDI region has the highest number of deaths. Although the region’s share of deaths declines slightly from 50% in 1990 to 48% in 2021, the actual number of deaths increases significantly in 2021. In contrast, high SDI regions and medium-high SDI regions have fewer deaths (Fig. 2). Among the GBD regions, South Asia accounts for more than half of the total global deaths, both in 1990 (59%) and in 2021 (58%). And India ranked first among 204 countries and regions, accounting for 51.10% of high-temperature-related self-harm deaths.

### 3.2 Global mortality trends and causes of the change

The decomposition analyses of high-temperature-related self-harm mortality across various dimensions globally in 1990 and 2021 showed a decline by 0.01 per 100,000 (Tables 1 & 2). By sex, females were the primary contributors to the decline in self-harm mortality with a reduction rate of 224.14%, while males had a negative contribution, with a rate of −126.34%. Changes in the population’s sex structure had a minimal effect on the decline in mortality, contributing only 2.20% (Table 1).

**Table 1:** Global high-temperature self-harm mortality decomposition by sex and age for 1990 and 2021.

**Classification**	**Mortality rate (per 100,000 population)**			**Demographic structure (%)**			**Contributed rate (%)**	
		
	**1990**	**2021**	**Difference**	**1990**	**2021**	**Difference**	**Demographic**	**Specific factors**
**Sex**								
Both	0.20	0.19	−0.01	100.00	100.00	0.00	2.20	97.80

Male	0.23	0.25	0.02	50.35	50.17	−0.18	5.46	−126.34
Female	0.16	0.13	−0.04	49.65	49.83	0.18	−3.26	224.14

**Age**								
All age	0.20	0.19	−0.01	100.00	100.00	0.00	−59.30	159.30

<65 years	0.19	0.18	−0.01	93.90	90.24	−3.66	83.59	118.44
≥65 years	0.33	0.29	−0.04	6.10	9.76	3.66	−142.89	40.86

**Table 2:** Global high-temperature self-harm mortality decomposition by SDI and GBD regions.

**Classification**	**Mortality rate (per 100,000 population)**			**Demographic structure (%)**			**Contributed rate (%)**	
		
	**1990**	**2021**	**Difference**	**1990**	**2021**	**Difference**	**Demographic**	**Specific factors**
**SDI regions**								
Global	0.20	0.19	−0.01	100.00	100.00	0.00	−176.32	276.32

High SDI	0.06	0.09	0.03	16.49	13.86	−2.63	24.36	−49.47
High-middle SDI	0.07	0.06	−0.01	19.94	16.52	−3.42	27.46	13.46
Middle SDI	0.17	0.15	−0.02	32.30	31.03	−1.27	25.95	82.77
Low-middle SDI	0.45	0.37	−0.08	21.78	24.34	2.56	−131.84	226.80
Low SDI	0.21	0.20	−0.01	9.40	14.16	4.76	−122.41	2.81
Other regions	0.09	0.10	0.01	0.09	0.08	−0.01	0.16	−0.04

**Geographic regions**								
Global	0.20	0.19	−0.01	100.00	100.00	0.00	−234.69	334.69

South Asia	0.56	0.47	−0.09	20.50	23.40	2.90	−187.40	264.22
Western Sub-Saharan Africa	0.25	0.28	0.03	3.62	6.21	2.59	−86.48	−16.22
Southeast Asia	0.21	0.16	−0.05	8.73	8.85	0.12	−2.74	54.00
North Africa and Middle East	0.11	0.13	0.02	6.36	7.90	1.54	−22.77	−20.97
Eastern Sub-Saharan Africa	0.07	0.09	0.02	3.58	5.40	1.82	−18.75	−8.86
East Asia	0.12	0.07	−0.05	22.83	18.66	−4.17	47.89	138.70
High-income North America	0.07	0.09	0.02	5.28	4.69	−0.59	6.01	−15.88
Other regions	0.05	0.06	0.01	29.11	24.90	−4.21	29.54	−60.29

SDI, socio-demographic index.

By age, demographic shifts negatively affected the decline in mortality. The increase in the proportion of individuals aged over 65 from 6.10% in 1990 to 9.76% in 2021 was the main contributor to this negative effect, contributing −142.89% (Table 1).

Demographic changes across SDI regions exhibited an negative impact on the global decline in mortality in the past 32 years, with a contribution rate of −176.32%. The largest negative impact came from the increasing population share of low-middle SDI regions, contributing −131.84%. However, changes in mortality rates within SDI regions had a positive impact on the global decline in mortality, with low-middle SDI regions contributing the most at 226.80%. The rise in mortality rates in high SDI regions negatively impacted the global decline, with a contribution rate of −49.47% (Table 2). Among geographical regions, South Asia contributed the most to the decline in global mortality over the past three decades, with a contribution rate of 264.22%. However, population growth in South Asia hindered the decline in mortality, representing the primary negative impact with a contribution rate of −187.40% (Table 2).

### 3.3 The associated factors

Table 3 shows the relationship between national indicators and high temperature-induced self-harm mortality. The mean VIF for all explanatory variables is approximately 2.71, with a range between 1.13 and 5.05. This is well below the common threshold of 10, indicating that the model does not suffer from multicollinearity. Model 1 evaluated the effect of raw national indicators and showed that diurnal temperature range, vapor pressure, mean temperature and the rate of population growth rate are all significantly positively associated with high temperature-induced self-harm (β > 0, p < 0.05). Conversely, population density, urban population growth rate, and number of physicians per 1000 people show a negative correlation (β < 0, p < 0.05). However, the p-values for rain days, PM2.5, percentage of urban population, life expectancy at birth, GDP, and GDP growth rate are not statistically significant, suggesting no clear association with the burden of high temperature-induced self-harm.

**Table 3:** National indicators’ impact on heat-related self-harm mortality from 1990 to 2021.

**Indicator**	**Model 1**			**Model 2**		
	
	**β (×10^−2^)**	**95% CI (×10^−2^)**	***P* value**	β*	**95% CI**	***P* value**
**Meteorology**						
Diurnal temperature range (°C)	1.870	1.334, 2.406	<0.001	0.223	0.159, 0.286	<0.001
Vapour pressure (hPa)	3.097	2.676, 3.519	<0.001	0.867	0.749, 0.985	<0.001
Rain days (days)	0.005	−0.009, 0.020	0.460	0.017	−0.028, 0.061	0.460
**Air pollution**						
PM2.5 (µg/m^3^)	0.008	−0.069, 0.085	0.842	0.007	−0.062, 0.076	0.842
**Demographic**						
Population density (people per sq. km of land area)	−0.007	−0.009, −0.006	<0.001	−0.571	−0.690, −0.452	<0.001
Population growth (annual %)	0.472	0.252, 0.692	<0.001	0.222	0.119, 0.326	<0.001
Urban population (% of total population)	0.038	−0.002, 0.079	0.062	0.036	−0.002, 0.074	0.062
Urban population growth (annual %)	−0.267	−0.462, −0.072	0.007	−0.157	−0.272, −0.042	0.007
**Socioeconomics**						
expectancy at birth, total (years)	−0.044	−0.097, 0.009	0.105	−0.031	−0.068, 0.006	0.105
GDP per capita growth (annual %)	−0.001	−0.023, 0.022	0.959	−0.001	−0.048, 0.045	0.959
GDP per capita (constant 2015 USD)	0.000	−0.000, 0.000	0.985	0.001	−0.054, 0.055	0.985
Physicians (per 1,000 people)	−0.630	−1.069, −0.191	0.005	−0.053	−0.090, −0.016	0.005

β, the beta coefficient; GDP, gross domestic product; USD, United States dollar. Fixed-effects model was used; Model.1 incorporates raw national indicator; Model.2 incorporates standardized national indicators.

Model 2 analysis revealed that standardized vapor pressure, population density, population growth rate, diurnal temperature range, urban population growth rate and the number of physicians per 1000 people had the greatest impact on high temperature-induced self-harm mortality. For each unit increase in their standardized units, the mortality rate (per 100,000 people) increased by 0.867, −0.571, 0.222, 0.223, −0.157 and −0.053, respectively. Overall, meteorological indicators had the most significant impact on the burden of self-harm due to heat exposure, followed by demographic indicators (Table 3).

## 4. Discussion

This study examined the causes and factors influencing changes in heat-related self-harm mortality due to high temperatures. Over the past 32 years, global self-harm mortality associated with high temperatures has declined, with significant contributions to this decline observed among females, in low-middle SDI regions, and in South Asia. In addition to meteorological factors, demographic indicators such as population density have been shown to impact the burden of high temperature-induced self-harm. However, global population aging and the growing populations in low-middle SDI regions and South Asia have added to the mortality burden, slowing the overall decline in global mortality rates.

South Asia accounts for 58% of global heat-related self-harm deaths but has also contributed the most to the 32-year decline in mortality rates. Over this period, South Asia experienced the fastest population growth globally, with a 2.90% increase in its share of the global population. This growth, however, has tempered its contribution to the global decline in mortality. India, the largest country in South Asia, has seen rapid population growth in recent decades [27]. Social inequalities linked to this growth can elevate suicide risks [28]. With its predominantly tropical monsoon climate and persistently high temperatures, India is particularly vulnerable. High ambient temperatures can affect the density of serotonin transporter proteins, leading to increased irritability and impulsivity [29]. The relationship between suicide and ambient temperature has been extensively studied. Both studies [30] with long-term data from a single country and studies [31] covering multiple countries and cities, albeit with regional and temporal variations, have shown that elevated ambient temperatures are generally associated with an increased risk of suicide. Additionally, climate change can exacerbate psychological issues such as anxiety and depression, further increasing the risk of self-harm [32]. As climate extremes become more frequent and severe in the coming years, there is an urgent need to address the global health threat posed by heat exposure.

The burden of heat-induced self-harm is unevenly distributed across SDI regions, likely due to socio-economic factors and varying levels of heat exposure. While low-middle SDI regions have significantly contributed to the global decline in heat-related self-harm mortality, the overall burden remains high. This may be attributed to the reliance of many in impoverished areas on agriculture and industry for their livelihoods—sectors particularly vulnerable to extreme weather events. Working in high-heat environments and facing economic instability from these events can heighten mental stress, increasing risks of anxiety, depression, and self-harm [32, 33].

In contrast, high SDI regions report the lowest burden of heat-induced self-harm, but this burden has shown a notable increase over the past 32 years. A key factor may be the rising youth suicide rates in these areas. Economic recessions, growing social inequality, gambling, and increased drug dependency have all been linked to higher rates of self-harming behavior among young people [34]. Additionally, online victimization has been positively correlated with self-harming behaviors [35]. The widespread use of the internet, growing reliance on social media, and susceptibility to harmful online content may contribute to the increasing burden of self-harm in high SDI regions.

Females have been the primary contributors to the global decline in mortality from heat-related self-harm. This may be linked to the increase in gender equality in many countries over the past 32 years. Evidence suggests that improvements in domestic gender equality are associated with reductions in suicide rates among females [36]. In contrast, global population aging has hindered the decline in heat-related self-harm mortality. Older individuals are more vulnerable to the mental health impacts of high ambient temperatures [37]. Additionally, stress, bereavement, low socio-economic status, and poor physical health can amplify the risk of self-harm among older adults during periods of high heat [38].

Numerous studies have examined the relationship between meteorological variables, air pollutants, and self-harm. A consistent positive association has been found between high temperatures and self-harm across studies [39]. However, associations between other meteorological factors—such as diurnal temperature difference, humidity, air pressure, rainfall days, and sunshine hours—and self-harm remain inconclusive [40]. This study found no significant correlation between PM2.5 and the burden of self-harm under heat exposure, possibly because the delayed and cumulative effects of air pollution on self-harm were not accounted for. Meteorological variables and air pollutants vary significantly by season, and self-harm itself is seasonal, peaking in spring [41]. High temperatures may influence biological responses to environmental factors [42], and there is evidence that high temperatures and air pollution may act synergistically to increase the risk of self-harm.

While the mechanisms linking air pollution and self-harm require further investigation, a growing body of research suggests a plausible connection. This study found that demographic indicators, including population density and urban population growth rates, influence the burden of self-harm from exposure to high temperatures. Specifically, the burden of heat-related self-harm was lower in areas with higher population densities and greater levels of urbanization. These findings align with previous research [13]. One explanation for the disproportionate impact of heat on communities with lower socio-economic status is their heightened vulnerability due to factors such as inadequate housing, limited access to air conditioning, poor health, and restricted access to healthcare [43]. Our findings support this hypothesis, as countries with a higher number of doctors had a lower burden of heat-related self-harm. Allocating more resources to healthcare services in the future could positively impact the prevention of self-harm. Furthermore, economic development improves thermal protection. For example, in cities where air conditioning is widely available, thermal risks are significantly reduced or eliminated [44]. And heat-related mortality has declined as air conditioning penetration has increased [45]. However, the study’s inclusion of urbanization indicators was limited, and other dimensions of urbanization were not explored. Further research is needed to better understand the relationship between urbanization and the burden of self-harm under high-temperature exposure. This study did not take into account some potential residual confounding factors that could affect the results. For example, the working environment (indoor or outdoor), the prevalence of air conditioning, the insulation of buildings, the amount of greenery and the prevalence of heat protection measures, etc. may affect the heat exposure of the population and to some extent affect the accuracy of the study results. Therefore, future studies should take these factors into account in order to assess the health effects of heat more comprehensively.

Based on the findings of this study, there is a need to allocate healthcare resources strategically in future - the burden of heat-related self-harm is higher in regions with lower SDIs. Policymakers should rationalize the allocation of healthcare resources based on demographic and economic conditions, tailoring interventions to meet local needs. It is important to incorporate climate and pollution factors into policies - Suicide prevention policies should consider the impact of air pollution and climate extremes, particularly high temperatures, on self-harm rates. In addition, it is crucial to prioritize physical and mental health in future - Countries must focus on promoting the physical and mental well-being of their populations to mitigate the risk of heat-related self-harm, especially in the context of global warming and urbanization.

It should be noticed there are four main limitations in this study: 1) The data on heat-related self-harm deaths obtained from GBD2021 are based on estimates of daily ambient temperatures, which are calculated with inherent limitations and do not take into account the delayed effects of high temperatures or interactions with other meteorological factors [22]. 2) The study examines only ecological associations between selected indicators and heat-induced self-harm, without delving deeply into the mechanisms underlying these relationships. This might limit the discussion of the fundamental factors driving the burden of heat-related self-harm. 3) Due to the univariate decomposition analysis used in this study, it is not possible to discuss in depth the interaction effects of gender, age, SDI region, and geographic region. 4) In panel models, although the variables have been standardized, the effects may differ across countries due to all the meteorological and environmental factors that may differ across countries and regions.

## 5. Conclusions

Declines in self-harm mortality due to high temperatures were primarily driven by females, low-middle SDI regions, and South Asia. However, global population aging and the growth of populations in these regions have increased the overall mortality burden, slowing the pace of decline in global self-harm mortality. Demographic factors, such as population density, are also associated with these trends. With rising exposure to environmental heat, it is urgent for countries to implement more targeted strategies to mitigate heat-related self-harm risk, ensuring interventions are tailored to local conditions and circumstances.

## Abbreviations

SDI: Socio-Demographic Index
GDP: gross domestic product
USD: United States dollar
CI: confidence interval

## Declarations

### Availability of data and materials

All data are openly accessible at VizHub - GBD Results (healthdata.org), Climatic Research Unit - Groups and Centres (uea.ac.uk) and World Development Indicators | Data Catalog (worldbank.org).
